# Supplementary material for: Application of simultaneous uncertainty quantification and segmentation for oropharyngeal cancer use-case with Bayesian deep learning
Source: Commun Med (Lond). 2024 Jun 8;4:110. doi: 10.1038/s43856-024-00528-5 (PMC11162474; doi:10.1038/s43856-024-00528-5)
Supplement: Supplementary file 2 — Supplemental Material [file 43856_2024_528_MOESM2_ESM.pdf]

Supplementary Methods

Additional details on the MDA external validation dataset are provided here.

For the 67 patients included, FDG-PET/CT scans were acquired from various GE Medical Systems scanners. Specifically, Discovery RX (n=27), Discovery STE (n=26), Discovery ST (n=12) and Discovery HR (n=2) models were used. Image acquisition parameters are shown in Supp Table 1. A 90-minute uptake period of rest was used for all patients. Attenuation corrected images were reconstructed using an ordered subset expectation maximization (OSEM) iterative algorithm (2 iterations, 18-24 subsets, 5mm Gaussian filter).

Demographic and clinical variables for the patients are presented in Supp Table 2. All data were extracted from clinical notes (EPIC electronic health record). Patients were staged using the seventh edition of the American Joint Committee on Cancer staging system. Most patients were males with base of tongue primary tumors. All patients were human papillomavirus positive.

Supplementary Results

Additional Uncertainty Measures and Performance Analysis

In this section, we present additional results for uncertainty quantification with mean surface distance (MSD) and Hausdorff distance at 95% (95HD) as well as additional performance analysis.

Instead of predicting the binary quality for the segmentation and model uncertainty based on the interobserver variability, in Supp Figure 1 and Supp Table 3 we illustrate the prediction of segmentation performance in terms of DSC value with a linear model where DSC is the dependent variable and uncertainty is the independent variable.
The segmentation and uncertainty quality thresholds used in Supp Figures 2-3 and Supp

Tables 4-5 are based on the interobserver variability value of 2.5 mm for MSD (as derived from clinician data in an analogous patient population [1]) and 6.5 mm for the 95HD (as derived from median HECKTOR participant benchmark data [2]).

The uncertainty thresholds for the 2.5 mm cross-validation MSD are at 2.6e-03 for EH, 3.6e-03 for H , 9.8e-04 for I, 1.4e-01 for CV, 3.8e-01 for SEH, 4.5e-01 for SH, 7.2e-02 for SI, and -8.0e-01 for R_{DSC} with MC Dropout Ensemble. For the Deep Ensemble 2.4e-03 for EH, 2.9e-03 for H, 4.9e-04 for I, 1.3e-01 for CV, 3.9e-01 for EH, 4.4e-01 for SH, 4.9e-02 for SI, and -8.3e-01 for R_{DSC}. The uncertainty thresholds for the 6.5 mm cross-validation 95HD are at 2.7e-03 for EH, 3.7e-03 for H , 1.0e-03 for I, 1.5e-01 for CV, 3.8e-01 for SEH, 4.6e-01 for SH, 7.6e-02 for SI, and -7.9e-01 for R_{DSC} with MC Dropout Ensemble. 2.5e-03 for EH, 3.0e-03 for H, 5.3e-04 for I, 1.4e-01 for CV, 3.9e-01 for EH, 4.4e-01 for SH, 5.4e-02 for SI, and -8.3e-01 for R_{DSC} with the Deep Ensemble.

In terms of processing time for auto-segmentation OPC GTVp, the method provides substantial improvements in comparison to manual segmentation as processing each scan takes on average in 2 and 8 seconds with Deep Ensemble and MC Dropout Ensemble, respectively, on a machine with RTX 2080 Ti while expert delineation can take from 30 minutes to two hours depending on the size and shape of the tumor (as noted by Outeiral et al. [3]). Using the proposed framework in Figure 1, when referring most uncertain cases to medical experts, while automating the rest, the method can improve the average performance over interobserver variability in terms of DSC while reducing the overall processing time when evaluated on this dataset.

Additional Qualitative Analysis

In this section, we present qualitative results for select cases in the MDA holdout dataset. Specifically, we describe our interpretations of model predictions and uncertainty maps relative to ground truth across multiple axial image slices, similar to how a case would be reviewed in the clinic. For simplicity, we only describe results of the MD Dropout Ensemble model. “High” and “low” values are relative to median values described in the main text (e.g., high DSC is greater than 0.61).

Case 1: Low performance, high certainty.

Here we describe a case with low DSC (0.43) but high certainty $(-SH = -0.43)$. This corresponds to a patient with a human papillomavirus positive base of tongue T2N0M0 tumor as per American Joint Committee on Cancer 7th edition [4]. Axial slice representations from superior to inferior slices for this case are shown in Supp Figure 4. As can be seen in the inferior slices (slice 54), there is initially a relatively large degree of uncertainty about the beginning of the prediction. Subsequently (slice 66), the model correctly predicts the tumor at the left base of tongue, with a simultaneous region of uncertainty appearing at the right base of tongue, likely secondary to the high PET signal causing a potential area of false positivity. This false positive PET signal is not ultimately included in the predicted segmentation mask, which in this case is seen as a desired outcome. More superiorly (slice 79), in terms of uncertainty and the resultant prediction, the model seems to have erroneously localized to the hyper-metabolic core of the primary tumor. Finally, at the most superior slices (slice 90), it is noted that there was metal streak artifact induced by dental hardware, which may have interfered with model inference and subsequent uncertainty quantification, as no prediction was generated. Main takeaways from this case include the model overemphasizing PET signal (which has been previously noted in PET/CT auto-segmentation models) which is also reflected in the resultant uncertainty measures. Moreover, the image artifact may also impact performance and uncertainty estimation.

Case 2: High performance, low certainty.

Here we describe a case with high DSC (0.64) but low certainty ($-SH = -0.5$). This corresponds to a patient with a human papillomavirus positive tonsillar T2N2M0 tumor as per American Joint Committee on Cancer 7th edition. Axial slice representations from superior to inferior slices for this case are shown in Supp Figure 5. In the inferior-most slices (slices 18-

27), uncertainty is noted near the larynx, likely a byproduct of high PET signal. As before, this false positive PET signal is not ultimately included in the predicted segmentation mask, which in this case is seen as a desired outcome. More superiorly (slice 61), the model begins to predict a segmentation on only the right side of the base of tongue, when in reality the ground-truth is a bilateral segmentation. Importantly, the model starts to note uncertainty on the contralateral part of the image, which is a desired outcome. As we move further superiorly towards the tonsils (slice 70) the tumor begins to exhibit an uncommon presentation (discontinuous fragment, bilateral in both tonsils), but the prediction better starts to approximate the ground-truth; the uncertainty previously demonstrated at the contralateral side (left) is still present but has now started to become included in the predicted segmentation. Continuing superiorly (slice 78), there is still high uncertainty in the discontinuous fragment but the model is able to generate a reasonable prediction, however the model eventually starts to generate an implausible prediction in an air space (slice 85) as the prediction begins to generate a bilateral segmentation erroneously. As with the previous case, towards the superior-most part of the image (slices 90-94), metal streak artifact induced by dental hardware may alter the predictions and uncertainty estimation; notably, the prediction ignores the false positive PET signal. Main takeaways from this case include uncommon tumor presentations (e.g., fragmentation of tumor from one continuous piece to two pieces) may present issues in generating prediction and uncertainty. Moreover, as before, image artifacts may impact predictions and uncertainty estimation.

Case 3: Contralateral uncertainty.

Here we describe an interesting case with high DSC (0.64) and high certainty ($-SH = -0.41$). This corresponds to a patient with a human papillomavirus positive tonsillar T3N2M0 tumor as per American Joint Committee on Cancer 7th edition. Axial slice representations from superior to inferior slices for this case are shown in Supp Figure 6. At the inferior slice (slice 60), the model generates the prediction correctly at the left tonsil but starts to note uncertainty at the contralateral tonsil. Subsequently, at the more superior slice (slice 70) the contralateral portion is revealed as part of the ground truth segmentation. The model is still uncertain about the area and ultimately does not include it as part of the prediction. In other words, the contralateral uncertainty indicates a false negative area that the model is uncertain about. In a clinical workflow this would correspond to an area the clinician could choose to further investigate.

Case 4: Nodal uncertainty.

Here we describe an interesting case with high DSC (0.71) and high certainty ($-SH = -0.44$). This corresponds to a patient with a human papillomavirus positive base of tongue T1N2M0 tumor as per American Joint Committee on Cancer 7th edition. Axial slice representations from superior to inferior slices for this case are shown in Supp Figure 7. In the inferior-most slice (slice 22) there is noted uncertainty in the area of high PET signal (likely spurious signal), which is not included in the prediction, which in this case is seen as a desired outcome. More superiorly (slices 61-70) a metastatic lymph node is present on the right side of the image; there is corresponding noted uncertainty about this area and it is ultimately not included in the prediction. The model is able to generate a prediction for the right base of tongue tumor without issues. Notably, as observed through the majority of other cases, metastatic lymph nodes are normally not considered by the model at all, likely due to

the often large geometric distances between the nodal metastases and the primary tumors. In this case the node exhibits features (i.e. high PET signal) in close proximity to the primary tumor, which could have led to the model uncertainty about this prediction.

Supplementary References

1. Cardenas, C. E. et al. Comprehensive Quantitative Evaluation of Variability in Magnetic Resonance-Guided Delineation of Oropharyngeal Gross Tumor Volumes and High-Risk Clinical Target Volumes: An R-IDEAL Stage 0 Prospective Study. *Int. J. Radiat. Oncol. Biol. Phys*. **113**, 426–436 (2022).

2. Andrearczyk, V. et al. Overview of the HECKTOR Challenge at MICCAI 2021: Automatic Head and Neck Tumor Segmentation and Outcome Prediction in PET/CT Images. in *Head and Neck Tumor Segmentation and Outcome Prediction* 1–37 (Springer International Publishing, 2022).

3. Rodríguez Outeiral, R. et al. Oropharyngeal primary tumor segmentation for radiotherapy planning on magnetic resonance imaging using deep learning. *Phys Imaging Radiat Oncol* **19**, 39–44 (2021).

4. Edge, S. B. & Compton, C. C. The American Joint Committee on Cancer: the 7th edition of the AJCC cancer staging manual and the future of TNM. *Ann. Surg. Oncol*. **17**, 1471–1474 (2010).

Supp Fig. 1: Additional correlation analysis between model segmentation performance as dependent variable and model certainty as independent variable.

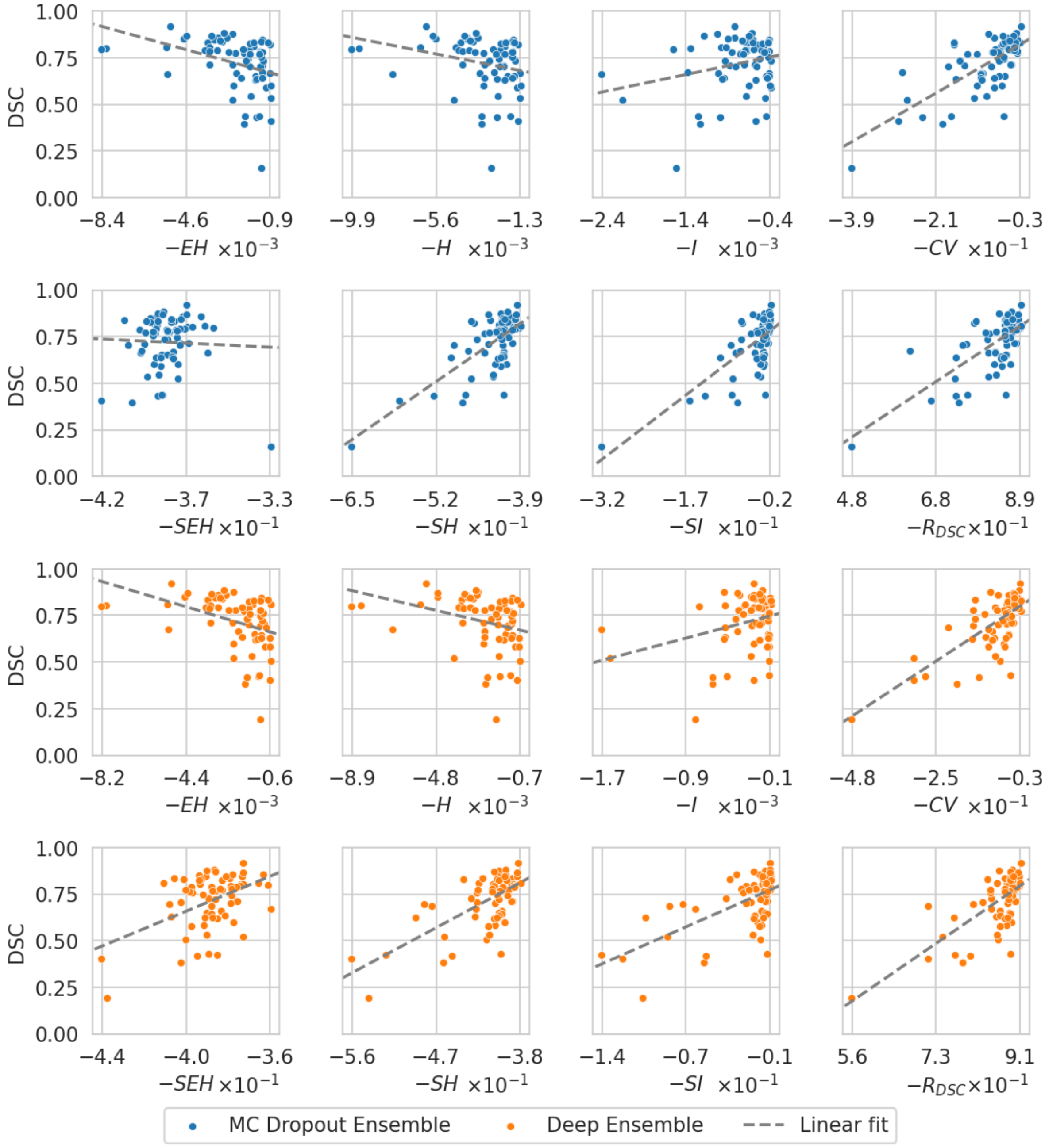

Segmentation performance is based on Dice similarity coefficient (DSC) in millimeters and model uncertainty based on expected entropy (EH), predictive entropy (H), mutual information (I), coefficient of variation (CV), structure expected entropy (SEH), structure predictive entropy (SH), structure mutual information (SI), and DSC-risk (R_{DSC}). In the linear fit, the DSC is the dependent variable and uncertainty is the independent variable. Note that the y-axis is shared between the rows.

Supp Fig. 2: Additional correlation analysis between model segmentation performance based on mean surface distance and model certainty.

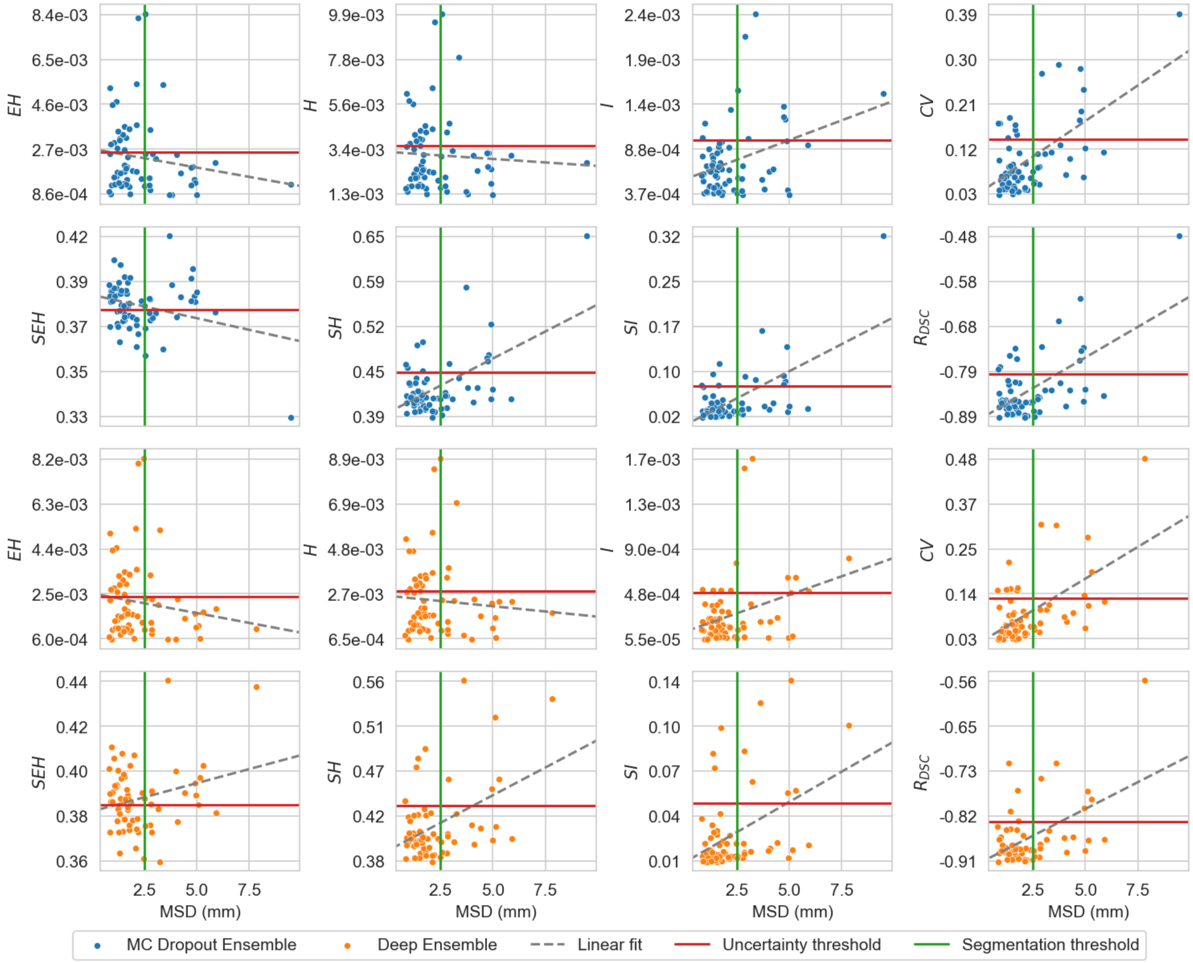

Segmentation performance is based on mean surface distance (MSD) in millimeters and model uncertainty based on expected entropy (EH), predictive entropy (H), mutual information (I), coefficient of variation (CV), structure expected entropy (SEH), structure predictive entropy (SH), structure mutual information (SI), and DSC-risk (R_{DSC}). The segmentation and uncertainty thresholds are drawn at the interobserver variability value of 2.5 mm MSD and at the predicted uncertainty value of the cross-validation 2.5 mm MSD, respectively. Note that the x-axis is shared between the columns.

Supp Fig. 3: Additional correlation analysis between model segmentation performance based on Hausdorff distance at 95% and model certainty.

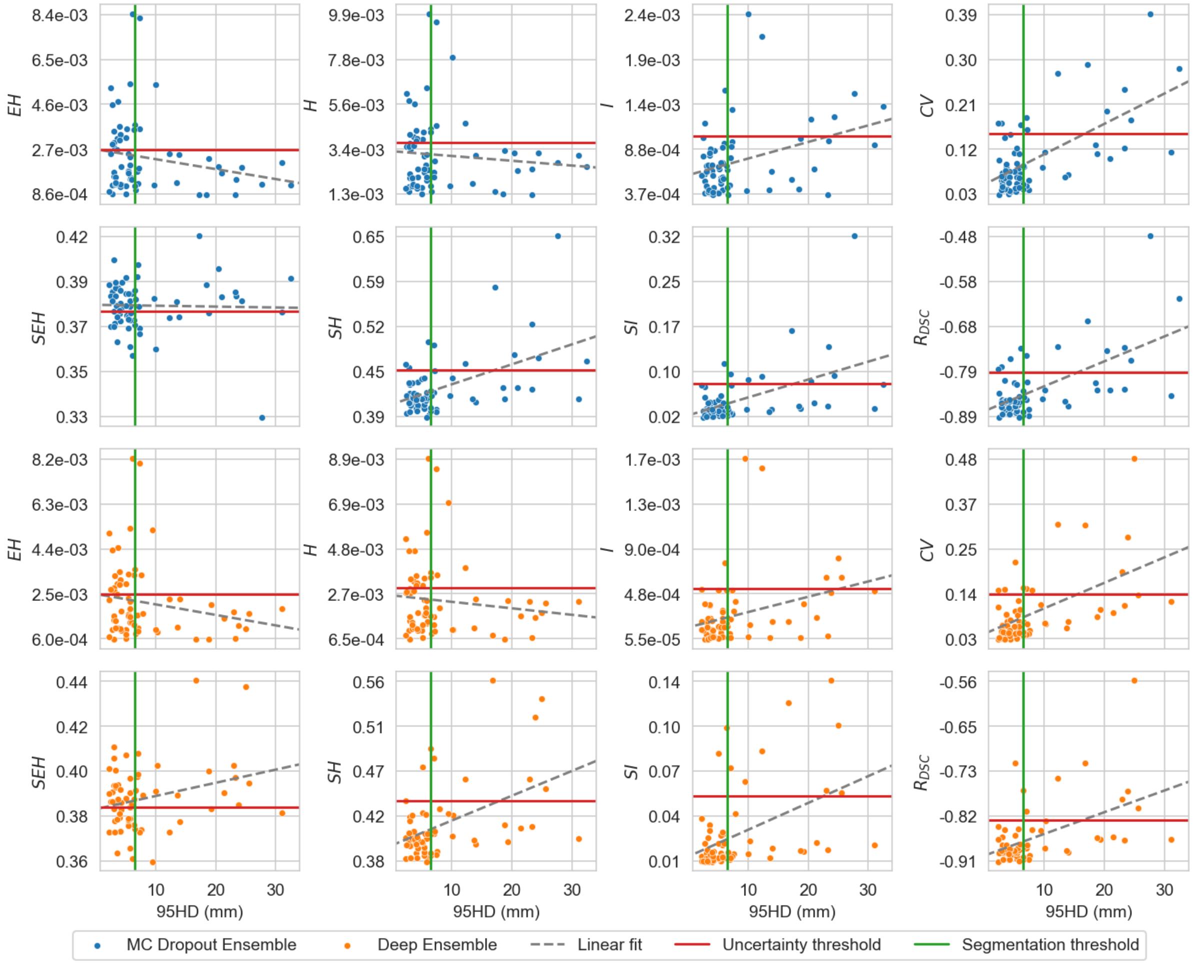

Segmentation performance is based on Hausdorff distance at 95% (95HD) in millimeters and model uncertainty based on expected entropy (EH), predictive entropy (H), mutual information (I), coefficient of variation (CV), structure expected entropy (SEH), structure predictive entropy (SH), structure mutual information (SI), and DSC-risk (R_{DSC}). The segmentation and uncertainty thresholds are drawn at the interobserver variability value of 6.5 mm 95HD and at the predicted uncertainty value of 6.5 mm cross-validation 95HD, respectively. Note that the x-axis is shared between the columns.

Supp Fig. 4: Additional qualitative investigation of a case with low performance and high certainty.

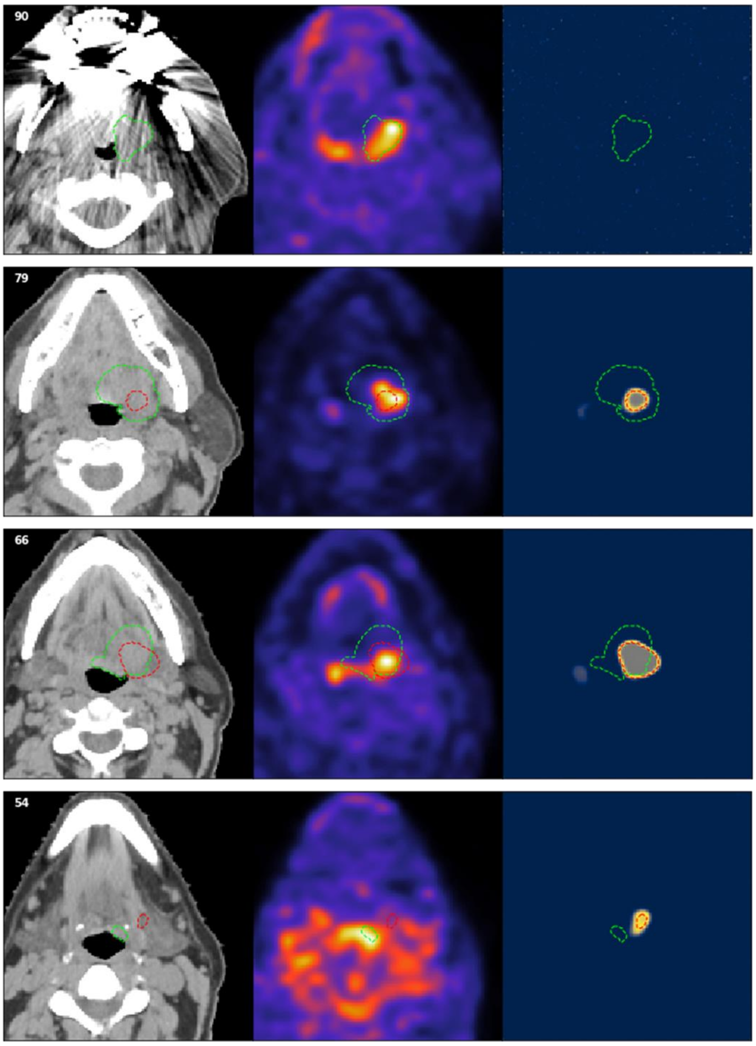

Number in top left corner = slice number; green dotted outline = ground-truth segmentation, red dotted outline = predicted segmentation. Blue, gray, and yellow colors in uncertainty maps correspond to low, medium, and high model uncertainty, respectively.

Supp Fig. 5: Additional qualitative investigation of a case with high performance and low certainty.

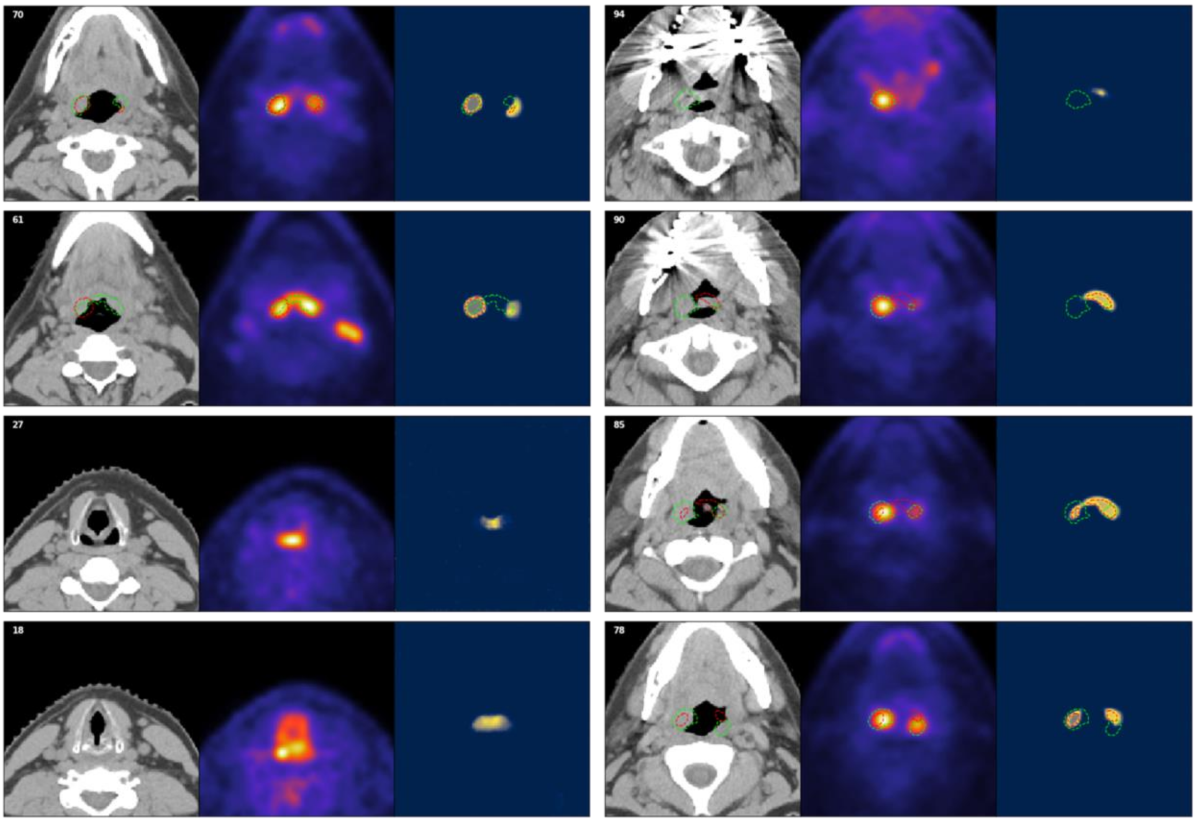

Number in top left corner = slice number; green dotted outline = ground-truth segmentation, red dotted outline = predicted segmentation. Blue, gray, and yellow colors in uncertainty maps correspond to low, medium, and high model uncertainty, respectively.

Supp Fig. 6: Additional qualitative investigation of a case with contralateral uncertainty.

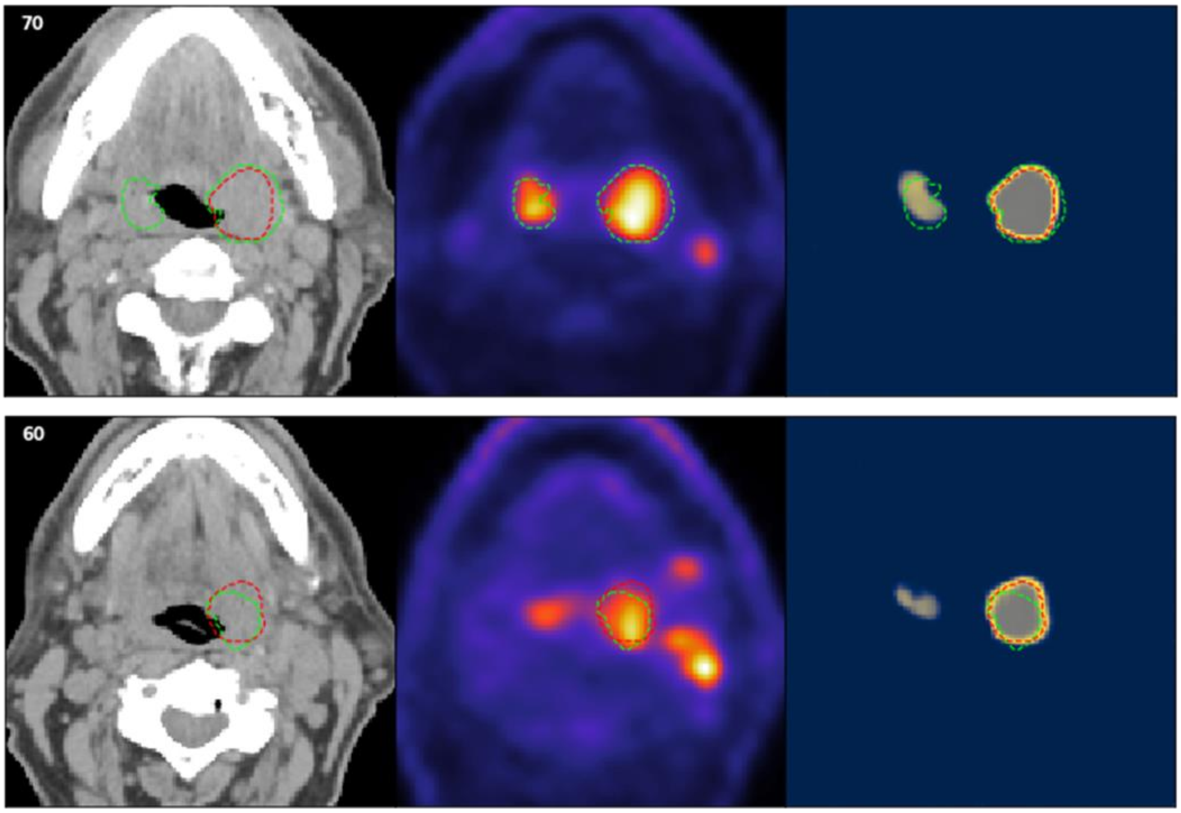

Number in top left corner = slice number; green dotted outline = ground-truth segmentation, red dotted outline = predicted segmentation. Blue, gray, and yellow colors in uncertainty maps correspond to low, medium, and high model uncertainty, respectively.

Supp Fig. 7: Additional qualitative investigation of a case with nodal uncertainty.

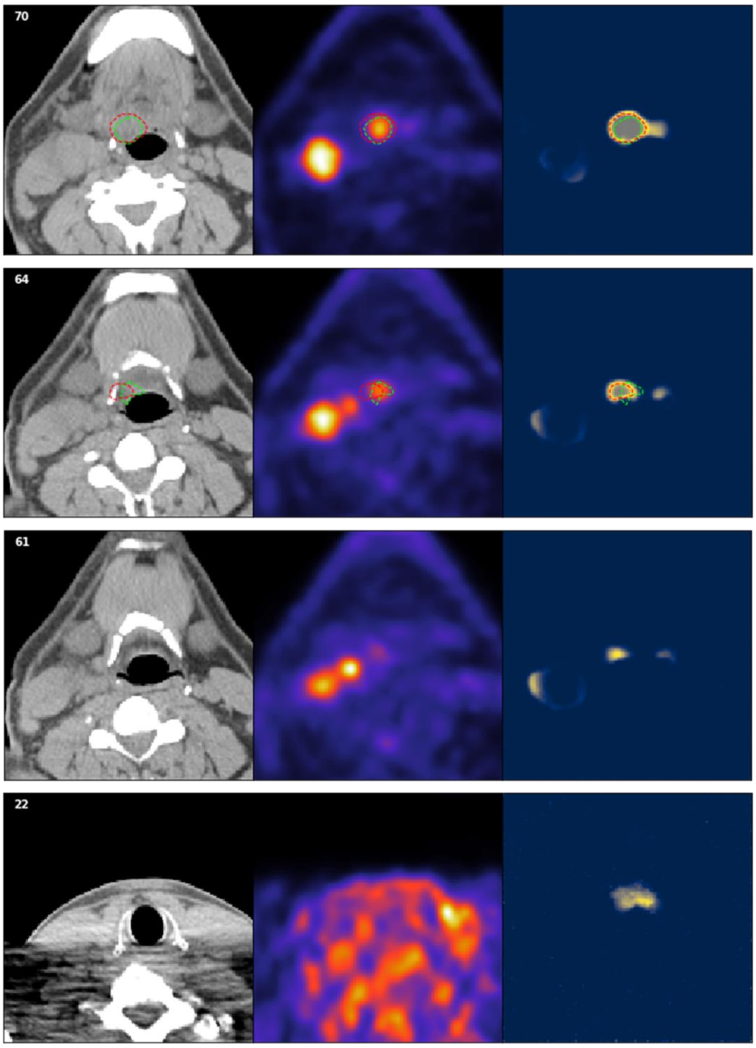

Number in top left corner = slice number; green dotted outline = ground-truth segmentation, red dotted outline = predicted segmentation. Blue, gray, and yellow colors in uncertainty maps correspond to low, medium, and high model uncertainty, respectively.

Supp Table 1: PET/CT image acquisition parameters for the MDA external validation dataset.

Acquisition Parameter	CT	PET
In-plane resolution (mm)	0.98	5.47
Slice thickness (mm)	3.75	3.27
Exposure time (ms)*	566 (500-676)	NA
X-ray tube current (mA)*	200 (100-296)	NA
KVP (kV)*	100 (100-120)	NA
Dose (Mbq)**	NA	375 (281-729)

All values were the same across all patients unless a parenthesis is shown, where the median and range are displayed. * only apply to CT data; ** only apply to PET data.

Supp Table 2: Demographic and clinical variable descriptive statistics for patients in the MD Anderson external validation cohort.

Variable	Value
**Age** (median, range)	57 (41-81)
**Self-identified Gender**	
Male	61
Female	6
**Subsite**	
Base of tongue	43
Tonsil	24
**T-Stage**	
T1	2
T2	34
T3	21
T4	10
**N-Stage**	
N0	8
N1	11
N2	48
**M-Stage**	
M0	61
M1	6
**Overall Stage**	
II	2
III	14
IV	51

Values shown are the absolute number of patients, except for age which is shown as the median and range. All patients were human papillomavirus positive and staged with the seventh edition of the American Joint Committee on Cancer staging system.

Supp Table 3: Correlation analysis of the linear fitting between model segmentation performance and model uncertainty.

**Model**	**MC Dropout Ensemble**		**Deep Ensemble**	
Uncertainty measure	Pearson correlation coefficient	p-value	Pearson correlation coefficient	p-value
EH	-0.361	2.7e-03	-0.388	1.2e-03
H	-0.258	3.5e-02	-0.303	1.3e-02
I	0.257	3.6e-02	0.303	1.3e-02
CV	**0.718**	7.9e-12	**0.720**	6.6e-12
SEH	-0.043	7.3e-01	0.465	7.2e-05
SH	0.704	3.0e-11	0.676	3.5e-10
SI	0.703	3.4e-11	0.623	1.8e-08
R_{DSC}	0.698	5.4e-11	0.704	3.1e-11

Pearson correlation coefficient and p-values for the linear fit model with Dice similarity coefficient (DSC) is the dependent variable and uncertainty is the independent value. Strongest correlations for each model are bolded.

Supp Table 4: Conditional probabilities and overall accuracy for mean surface distance based on uncertainty.

**Model**	**MC Dropout Ensemble**			**Deep Ensemble**		
Uncertainty measure	p(accurate| certain) (%)	p(inaccurate| uncertain) (%)	AU (%)	p(accurate| certain) (%)	p(inaccurate| uncertain) (%)	AU (%)
EH	58.7	14.3	44.8	60.9	9.5	44.8
H	62.5	21.1	50.7	63.8	15.0	49.3
I	**75.0**	**72.7**	**74.6**	75.0	54.5	71.6
CV	71.7	50.0	67.2	74.5	50.0	70.1
SEH	63.0	30.0	43.3	74.1	32.5	49.3
SH	72.7	58.3	70.1	75.4	60.0	73.1
SI	74.5	66.7	73.1	**77.2**	**70.0**	**76.1**
R_{DSC}	72.7	58.3	70.1	75.4	60.0	73.1

Accurate/inaccurate is determined by the 2.5 mm MSD threshold and certain/uncertain is determined by the predicted confidence threshold from 2.5 mm validation MSD. The overall segmentation accuracy is defined as the accuracy vs uncertainty (AU). Best results for each model are bolded.

Supp Table 5: Conditional probabilities and overall accuracy for Hausdorff distance at 95% based on uncertainty.

Model	MC Dropout Ensemble			Deep Ensemble		
Uncertainty measure	p(accurate| certain) (%)	p(inaccurate| uncertain) (%)	AU (%)	p(accurate| certain) (%)	p(inaccurate| uncertain) (%)	AU (%)
EH	58.7	14.3	44.8	56.5	14.3	43.3
H	63.3	22.2	52.2	61.2	22.2	50.7
I	74.1	77.8	74.6	70.5	**83.3**	71.6
CV	**76.4**	75.0	76.1	71.4	63.6	70.1
SEH	68.0	33.3	46.3	**74.1**	40.0	53.7
SH	75.0	72.7	74.6	71.9	70.0	71.6
SI	75.9	**88.9**	**77.6**	73.7	80.0	**74.6**
R_{DSC}	74.5	66.7	73.1	72.4	77.8	73.1

Accurate/inaccurate is determined by the 6.5 mm 95HD threshold and certain/uncertain is determined by the predicted confidence threshold from the cross-validation 6.5 mm 95HD. The overall segmentation accuracy is defined as the accuracy vs uncertainty (AU). Best results for each model are bolded.
